# Perceptions About Cannabis Following Legalization Among Pregnant Individuals With Prenatal Cannabis Use in California

**DOI:** 10.1001/jamanetworkopen.2022.46912

**Published:** 2022-12-14

**Authors:** Kelly C. Young-Wolff, Tara R. Foti, Andrea Green, Andrea Altschuler, Monique B. Does, Melanie Jackson-Morris, Sara R. Adams, Deborah Ansley, Amy Conway, Nancy Goler, Maha N. Mian, Esti Iturralde

**Affiliations:** 1 Division of Research, Kaiser Permanente Northern California, Oakland; 2 Department of Psychiatry and Behavioral Sciences, University of California, San Francisco; 3 Sacramento Medical Center, Kaiser Permanente Northern California, Sacramento; 4 Regional Offices, Kaiser Permanente Northern California, Oakland

## Abstract

**Importance:**

As rates of prenatal cannabis use increase and cannabis legalization spreads across the US, studies are needed to understand the potential impacts of legalization from the perspectives of pregnant individuals who use cannabis.

**Objective:**

To characterize pregnant individuals’ perspectives on legalization of cannabis for adult use in California (effective in 2018) in relation to prenatal cannabis use behaviors and beliefs.

**Design, Setting, and Participants:**

This qualitative study was conducted in Kaiser Permanente Northern California, a large health care system with universal screening for self-reported cannabis use at entrance to prenatal care. Eighteen semistructured focus groups were conducted from November 17 to December 17, 2021, using a secure video conferencing platform with Black and White pregnant participants who self-reported cannabis use during early pregnancy. Data were analyzed from March to June 2022.

**Main Outcomes and Measures:**

Video-recorded interviews were transcribed and analyzed using thematic analysis to identify major themes and subthemes.

**Results:**

Among 53 participants (mean [SD] age, 30.3 [5.2] years), 23 (43%) identified as non-Hispanic Black and 30 (57%) identified as non-Hispanic White; 16 participants (30%) reported continued cannabis use at the time of recruitment. Major themes regarding the perceived impact of legalization included easier access (via retailers and delivery), greater acceptance (including reduced stigma and more discussions about prenatal cannabis use with health care practitioners), and trust in cannabis retailers (including safety and effectiveness of diverse products sold and perceptions of cannabis retailer employees as knowledgeable, nonjudgmental, and caring). Responses were mixed about whether retailer marketing and advertising were associated with prenatal cannabis use and whether legalization resulted in reduced concerns about Child Protective Services involvement.

**Conclusion and Relevance:**

The findings of this qualitative study suggest pregnant individuals perceive cannabis legalization as having reduced barriers to prenatal cannabis use and that legalization has created challenges and opportunities for supporting the health of pregnant individuals. The results of this qualitative study highlight key areas that can be further explored in future educational materials, public health campaigns, and policy adaptations to address increasing rates of prenatal cannabis use.

## Key Points

**Question:**

Is cannabis legalization for adult use associated with cannabis use behaviors among pregnant individuals?

**Findings:**

This qualitative study of 53 pregnant individuals who used cannabis found consistent beliefs that legalization led to easier cannabis access (via retailers and delivery), greater acceptance (including reduced stigma, more patient-clinician discussions about prenatal cannabis use, and fewer concerns about Child Protective Services involvement), and trust in cannabis retailers (including safety and effectiveness of diverse products sold and perceptions of employees as knowledgeable, nonjudgmental, and caring).

**Meaning:**

These findings suggest that pregnant individuals perceive legalization as having reduced barriers to prenatal cannabis use, which creates challenges and opportunities for supporting the health of pregnant individuals.

## Teaser

This qualitative study characterizes pregnant individuals’ perspectives on whether legalization of cannabis for adult use in California in relation to prenatal cannabis use behaviors.

## Introduction

Cannabis use during pregnancy is associated with some adverse fetal outcomes, including low birth weight, and potential neurodevelopmental consequences for offspring exposed to cannabis in utero.^1,2,3,4,5,6^ Due to concerns about potential health risks, national organizations recommend that pregnant individuals abstain from using cannabis during pregnancy.^2,5^ Yet, the prevalence and frequency of prenatal cannabis use continue to increase in the US,^7,8^ and pregnant individuals report using cannabis to treat pregnancy-related symptoms, such as depression, stress, pain, problems with sleep, and nausea.^9,10,11^

As of May 27, 2022, all but 3 states have legalized some form of medicinal cannabis, and 19 states have legalized cannabis for adult recreational use.^12,13^ Legalization is associated with increased access and acceptability of cannabis^14,15,16^ and has contributed to expansion of alternative modes of administering cannabis (eg, vaping, ingestion of edible products), higher potency products, and extensive product diversification.^17^ Existing studies provide inconclusive evidence about whether state adult-use cannabis legalization is associated with changes in prenatal cannabis use.^18,19,20^ However, some cannabis retailer staff (sometimes called *budtenders*) promote cannabis as safe and effective for managing pregnancy symptoms,^21^ and greater cannabis retailer density around pregnant individuals’ homes is associated with higher odds of prenatal cannabis use.^22^

Understanding the impact of cannabis legalization from the perspective of pregnant individuals living in states where cannabis is fully legal is important to inform future educational materials, public health campaigns, and policy adaptations to address increasing rates of cannabis use during pregnancy. This focus group study explores perceptions of pregnant individuals regarding legalization of cannabis for adult-use and cannabis use behaviors and beliefs during pregnancy.

## Methods

### Design, Setting, and Participants

This qualitative study was conducted in Kaiser Permanente Northern California (KPNC), a large integrated health care delivery system serving more than 4 million diverse members.^23^ The KPNC Institutional Review Board approved this study. All participants provided verbal informed consent. This study followed the Consolidated Criteria for Reporting Qualitative Research (COREQ) reporting guideline for qualitative research.

California legalized cannabis for adult use (age ≥21 years) on November 9, 2016, with legal sales effective on January 1, 2018. Pregnant adults (aged ≥18 years) who identified as non-Hispanic Black or non-Hispanic White, based on their self-reported electronic health record data, and self-reported prenatal cannabis use on the universal prenatal screening questionnaire on entrance to prenatal care (at approximately 8 weeks’ gestation) were eligible for inclusion. We focused on Black and White patients in this initial study because they have had the highest prevalence of prenatal cannabis use in our health care system in recent years,^24^ and legalization may be associated with worsening of existing health and social disparities in maternal, child, and child welfare outcomes.^25^

Patients who reported daily or weekly prenatal cannabis use on the questionnaire were prioritized. Electronic health record reviews were conducted to confirm that patients were still pregnant (ie, no documented pregnancy loss), and potential participants were sent an email describing the study with an option to opt out. Patients were then contacted by phone within 1 week of the email, invited to participate, and consented. Patients were informed that their clinical care would not be impacted by their participation. During recruitment, patients were asked whether they were currently using cannabis.

Of 304 identified eligible patients, 200 (66%) were not scheduled for focus group participation (139 were unable to be reached, 53 refused, 2 were found to be ineligible, 5 had time conflicts, and 1 did not complete the consent process). Of 104 individuals (34%) who had a focus group scheduled, 53 (51%) participated in 1 of eighteen 90-minute focus groups (1-6 participants per group), including 8 groups with Black participants (23 participants total) and 10 with White participants (30 participants total). Focus groups were completed during a 4-week period, and scheduling and rescheduling was not possible for many individuals who consented. Focus group leaders and participants were matched on race and ethnicity, enhancing congruency between participants and researchers. This strategy was chosen given evidence that people with more shared experiences may be more open with one another,^26,27^ and to acknowledge the role that race and ethnicity play in the experiences of pregnant individuals. Of 51 scheduled individuals (49%) who did not complete the focus group, 39 did not show up, 10 canceled, and 2 had groups that were canceled by the group leader.

### Focus Group Guide

A semistructured focus group guide was developed by the study team. Question domains included drivers of prenatal cannabis use, harms and benefits, changes in use during pregnancy, modes of administration, and communication with clinicians. The semistructured format allowed for new themes to emerge during focus groups.

### Data Collection

Focus groups took place virtually over 4 weeks (November 17 to December 17, 2021) via Microsoft Teams, a Health Insurance Portability and Accountability Act–adherent video-conferencing platform, and were facilitated by 2 members of the study team (T.R.F. and A.G.). Participants were encouraged but not required to have their camera on during the focus group. Team members met regularly to review field notes and discuss preliminary themes. All agreed that thematic saturation was achieved after the 18th focus group. Groups were recorded and deidentified and then professionally transcribed. Participants received a $50 gift card following focus group completion.

### Qualitative Analysis

We analyzed the transcripts using a thematic analysis approach. Three experienced, independent coders reviewed all of the transcripts (K.Y.W., T.R.F., and A.A.) and created a draft codebook. Reviewers (K.Y.W., T.R.F., A.A., M.B.D., and E.I.) independently coded 2 transcripts to test the codebook and further refine it until consensus was achieved on themes and subthemes. Transcripts from the 16 remaining focus groups were manually coded by the study team using NVivo qualitative analysis software release 1.6.1, and quotes were selected from transcripts according to themes and subthemes.

We compared transcripts for differences in themes and subthemes by participant race. *P* values were 2-sided, and statistical significance was set at *P* < .05. Data were analyzed from March to June 2022.

## Results

A total of 53 participants were recruited, and at the time of recruitment, participants had a mean (SD) age of 30.3 (5.2) years; 23 participants (43%) identified as non-Hispanic Black and 30 participants (57%) identified as non-Hispanic White. Based on self-report by the participants on their prenatal screening questionnaire, a total of 37 participants (70%) reported daily prenatal cannabis use, 13 participants (25%) reported weekly use, and 3 participants (6%) reported monthly or less use; 37 participants (70%) reported that they had stopped cannabis use at the time of recruitment. There were 9 participants (17%) in their first trimester, 25 participants (47%) in their second trimester, and 19 participants (36%) in their third trimester.

Among the 304 individuals contacted, Black individuals were more likely to schedule a focus group compared with White individuals (56 individuals [42%] vs 48 individuals [28%]; *P* = .008). The 104 scheduled and 200 not-scheduled individuals did not differ on age, weeks pregnant, or frequency of prenatal cannabis use. Among the 104 women who were scheduled for a focus group, 53 participants and 51 nonparticipants did not differ on age, weeks pregnant, frequency of cannabis use, trimester at recruitment, or current cannabis use at recruitment; however Black individuals who scheduled were less likely to participate than White individuals (23 individuals [41%] vs 30 individuals [63%]; *P* = .03).

We identified 3 main themes related to cannabis legalization across all interviews. No differences in these themes were identified by participant race. Themes and subthemes are shown in Table 1 along with illustrative quotations from participants.

**Table 1.** Themes, Subthemes, and Exemplar Quotes About the Perceived Impacts of Cannabis Legalization for Adult Use

Theme and subthemes	Salient quote
**Greater access and exposure to cannabis**	
Retailer availability	“You look at the cannabis clubs on the corners, that it’s literally like a liquor store here. You know, the easy access, the convenience.”
	“I feel like it’s like corner stores. Like, you know how they say in like in low-income neighborhoods, like, they always have corner stores instead of Whole Foods or something like that? And I feel like the more we see cannabis clubs like the more people are gonna want to go to ‘em or use it, you know, like if they was going to the corner store and getting junk food.”
	“I think now weed is easier to get more than ever, probably easier to get than cigarettes.”
Convenience and discretion of delivery	“I think people are smoking more now because there are deliveries where people are not actually going to the actual dispensaries, but it’s being delivered to their house either for free or for a deal. So, the fact that it’s being dropped off to you, I think, makes a big difference. It’s a little more discreet as well.”
	“It’s just a lot safer and easier and more convenient to go for a delivery….I’m definitely in a different stage of my life. Like, I’m not trying to find somebody’s cousin’s brother’s nephew who can give me something. I like to kind of know where my stuff comes from also. You know, inside or outside of pregnancy.”
Widespread marketing and advertising	**“**When I see the billboards, it just makes me, like, miss it….I’d literally drive by it every day. And, you know, that was my go-to on the way home. It was just, like, a stop. Or I see the advertisements for Eaze [cannabis delivery service] on the billboard…it makes me miss it, because I’m definitely not doing it as frequently as I was before. And then you get the text messages, or they’re having this [sale]…it’s kind of hard to see. It’s like trying to stay away from something, but then it’s kind of, like, just in your face a little bit.”
	“So seeing them [billboards and advertisements] all the time, I’m like, ‘damn, I wish but, you know, not right now.’ So, but it’s definitely enticing. But I don’t think it would sway me either way if that makes sense.”
	“I get text messages from one of the dispensaries that I go to almost every day of, like, their deal of the day, and I always get pissed off. I’m always like, ‘screw you. You know I can’t [use cannabis during pregnancy].’”
**Increased acceptance**	
Reduced stigma	“It’s legal. Like 5 years ago like if you smoked weed while pregnant, people would be looking at you like, ‘you’re crazy.’ Like, ‘what are you doing?’ They’re like, ‘who sold a pregnant person some weed?’ Now, it’s like you might see a group of pregnant people just passing the blunt around. Like, ‘okay,’ like, ‘y’all smoking, okay.’”
	“You can just go to the cannabis club now. Like pregnant or not, as long as you’ve got your ID, like, they’ll let you go. I feel like before I stopped, before I found I was pregnant, this girl was like 8 months, I said, ‘you’re going to buy weed?’ She was like, ‘yeah. It’s legal.’”
	“I think the stigma is slowly becoming… not as bad… in the state of California, it’s legalized, so I think that’s also a part of it. I think because people are more willing to talk about it and be open about it… it makes me feel good when I hear there are other people that, you know, still are using while they’re pregnant.”
More discussions with health care providers	**“**[In my first pregnancy, before cannabis was legal], I was like ‘oh, yeah I smoke, but I don’t even really smoke that much,’ but really and truly, I probably was smokin’ every day, you know? So, but now, I just told my doctor, like, ‘yeah, I smoked a joint, like, last week because I was feeling anxious, whatever, and they allowed more understanding, where like before, if I tell ‘em, then they gon’ look down on me and I’m gon’ feel bad about it, and, like, I don’t feel bad about it now.’”
	“I think that if it wasn’t legal and I didn’t feel comfortable speaking about it in the beginning with my doctor… I may still be smoking because if I didn’t say anything and didn’t have those conversations with my doctor, I probably would have just kept doing it.”
	“Whereas if you’re honest about it, the doctor is going to be like, ‘okay, she’s been honest with me this entire pregnancy. I know this is what she has chosen for this and this and this.’ You know what I mean? Even if they chose it for recreational uses, I think that they are understanding about it, they know that it’s recreationally legal, so why put down a mother for choosing what goes in her and her own child’s body?”
Mixed concerns about Child Protective Services (CPS)	“I’ve heard people say, you know, if they find marijuana in your blood when you’re having your baby, that they’re gonna take your baby from you at the hospital and make you go through CPS and stuff like that, and that was kind of a concern. Not so much now for me, because of the world we live in and the fact that we live in California, but at some point before 2020, it was definitely a concern of them testing you for marijuana, then taking your baby.”
	“I definitely think that the legalization of it, you know, definitely takes away a lot of the stigma for women and the fear of getting your child taken away.”
	“[To my physicians], I’m like, you know, ‘I’m still using cannabis. Is CPS going to be called?’ They’re like, ‘absolutely not. CPS will not be called unless you’re using, like, heroin.…And so it’s definitely, absolutely, 110% due to it being legalized.”
	“What pushes women to be secretive about it’s all been documented in your medical records, and to have somebody be like, ‘well, you were smoking throughout your pregnancy,” and for them to feel like you’re putting your child at risk and especially in the environment that we’re in now, to where we see these people taking people’s kids away for whatever reason through social services and stuff like that.”
	“I actually had a friend who has a CPS case out on her because she had tested positive for weed when she gave birth. So, that’s definitely a worry because some people are so against it. I don’t know if that’s in every case if that would happen. It’s kind of scary, you know?”
**Trust in cannabis retailers**	
Trust in cannabis product safety	“I got a 17-year-old, so I smoked when I was pregnant with her, and it’s probably a better quality of weed that I’m smoking than I was when I was smoking with her because then, I was buying from the streets.”
	“I was getting it from a dispensary just because I liked knowing that it was tested and where it was coming from. But I know people that get it off the streets just to get it a little bit cheaper. But I would hope that they, if they were going to use it during pregnancy, that they would get it from a known source just because of pesticides and how it’s grown and that kind of stuff.”
Diverse product selection	“I’m all about the investment that we have now where we have percentages, and we know more about our THC. So, I like choosing certain amounts of things. I definitely prefer my indica over sativa [cannabis type] because sativa gives me more anxiety.”
	“But I would probably prefer dispensaries, just because they have things that are grown specifically for certain things that are going on with you [during pregnancy], whether it be sleep or anxiety or, you know, appetite, or inflammation, or anything like that.”
	“The cannabis clubs come with different products of getting you high to where you’re not having to just smoke. If you’re not somebody who likes to smoke, you can go get drops that can you put into your tea and then you can still get the different side effects.”
Cannabis retailer staff as knowledgeable	**“**And a lot of the—those people [budtenders], they, they’re aware of what cannabis does for you at the dispensary, so if you walk in there pregnant like they’re going to be like, ‘Well, this is what’s best for you.’ They’re not gonna be like, ‘oh, you shouldn’t do it.’ They’ll actually show you what would be best for you at your—during your pregnancy, depending on your symptoms.”
	“I like the fact that, like, if I use something and then I don’t like it or whatever my experience, I could just go and talk to them and say, ‘okay, well, this is what this was, and this is what I don’t like,’ and they say ‘okay, well, here’s something new.’ You know like we go to a dealer on the street and they, they just got whatever, so it’s like, I don’t know. I feel more comfortable speaking to somebody that’s, like, I don’t know if they expert on weed but I know they know a lot more about the different strands than people do on the street.”
	“If you can find educational dispensaries, then those people know what they’re talking about, like they’re trained to be giving you that information because they are dealing with medicine. So, don’t just ask your friend on the street, ‘hey, do you think that’s a good idea?’ Ask somebody that is in the industry that has been in the industry or has been educated on the products that they’re talking to you about.”
Cannabis retailer staff as caring and nonjudgmental	**“**I personally feel like when you go to the dispensary, they actually care about you. When you go to the street, they’re not really worried about you feeling better. They’re more worried about the money…”
	**“**Yeah. It’s like not being able to go to your, like, regular grocery store anymore. Like, ‘oh, I miss having those conversations with those people that I saw on a regular basis.’ Or, like, a restaurant, I guess. Kind of like a restaurant.”
	“I have been going to the same dispensary for a while now. I told them I was pregnant because I was excited and they were all, I didn’t feel judgment from any of them….I don’t really have an issue going into a dispensary, but it’s the same one, so they know me. It’s like a bar for me, you know, if I drink. They all know me and my name.”

Abbreviations: CPS, Child Protective Services, THC, tetrahydrocannabinol.

### Greater Access and Exposure to Cannabis

#### Retailer Availability

Participants perceived that cannabis use among pregnant individuals they knew (eg, family members and friends) had increased in recent years in part due to easier access following legalization. They described ubiquitous cannabis retailers in their neighborhoods and believed that easy access and convenience increased desire to use cannabis. Several participants reported that cannabis retailers were as accessible as liquor stores or corner stores and compared the ease of accessing cannabis to getting cigarettes or alcohol. Participants did not differentiate medical vs adult-use retailers.

#### Convenience and Discretion of Delivery

Participants expressed the belief that widespread delivery options for cannabis following legalization contributed to increases in prenatal cannabis use. Many participants described delivery as convenient, more discreet, and safer than buying cannabis outside the legal marketplace. Other participants noted that delivery was often free, making this an especially easy option for obtaining cannabis.

#### Widespread Marketing and Advertising

Participants reported varied experiences with cannabis marketing and advertisements. Many participants who had quit or cut down on use during pregnancy described the enticing effects of seeing billboards and advertisements, reporting that it made them miss using or think about cannabis more. However, other participants reported that marketing and advertisements had little impact on their cannabis use behavior. Several who continued to use cannabis had set routines of purchasing products at regular intervals from their favorite retailers and reported that advertisements and other marketing strategies did not sway them. Participants expressed mixed responses to receiving text messages with promotions and deals during their pregnancy, ranging from “it hasn’t, like, made me go get some” and “doesn’t really affect me that much” to “screw you. You know I can’t [use cannabis while pregnant].”

### Increased Acceptance

#### Reduced Stigma

Most participants perceived notable postlegalization decreases in stigma associated with cannabis use in general and during pregnancy. Many participants emphasized the belief that reduced stigma contributed to increases in prenatal cannabis use. Several participants emphasized that parents or grandparents who had never used cannabis before it was legal now enjoy it, and this has led to greater acceptance of participants’ prenatal cannabis use by their families during their current pregnancy vs prior pregnancies before legalization. Some participants expressed doubts about whether prenatal use has actually increased following legalization and believed that pregnant individuals are now more able to talk openly about it.

#### More Discussion With Health Care Practitioners

Participants described greater confidence and willingness to discuss their use of cannabis during pregnancy with their obstetric health care practitioner as a result of legalization. One observed “I was very upfront [with my physician] because, you know what, it’s legal. What are they going to do?” Another noted her willingness to tell her physician because, “A lot of people do it and it’s not that big of a deal—it’s not illegal.” Many perceived their health care practitioners to be more understanding and less judgmental about their prenatal cannabis use after legalization. One participant reported that she was willing to disclose and honestly discuss her use of cannabis with her obstetrician because cannabis is legal, which resulted in her ultimately quitting use during pregnancy.

#### Mixed Concerns About Child Protective Services

Many participants explained that legalization contributed to reduced concerns about Child Protective Services (CPS) investigations. One described getting reassurance from her health care practitioner that CPS would not be called as a result of her prenatal cannabis use, and she attributed this to the impact of legalization. However, it is important to note that even after legalization, several participants reported continued strong concerns about potential CPS involvement after delivery.

### Trust in Cannabis Retailers

#### Trust in Cannabis Product Safety

Participants described greater trust in the quality and safety of cannabis products at retailers compared with buying it on the illicit market. Many preferred purchasing their cannabis from retailers because of their reliability, testing, and comfort knowing where and how it was grown. One participant noted, “I like to know what I’m getting, and, for me, that’s healthy and safe. I’m not going to go to Joe Blow off the street, because that’s sketchy.”

Notably, participants reported that the warnings about health risks on blunts (ie, cannabis rolled in tobacco leaf) sold at retailers contributed to greater perceptions of retailer legitimacy and safety. One participant described learning about the harms of smoking blunts during pregnancy from the mandated warnings on prenatal use of tobacco products; this led her to switch from wrapping her cannabis in tobacco leaves to nontobacco papers. Warning labels on use of other cannabis products during pregnancy were not discussed.

#### Diverse Product Selection

Participants appreciated the diversity of products available at cannabis retailers after legalization. Many noted the wide selection of products grown specifically for treating different mental health or medical conditions. Others appreciated the array of products with different modes of administration and reported that this increased accessibility for individuals who want to use during pregnancy but do not like smoking. One participant noted that product diversification could contribute to greater use, stating “I think the different options at the dispensary do make it, I don’t want to say tempting, obviously, but it’s like with more options, comes more hypothetical solutions.”

#### Cannabis Retailer Staff as Knowledgeable

Many participants viewed budtenders who sell cannabis in retail outlets as experts on the benefits of cannabis, including during pregnancy. They trusted staff to recommend products that were helpful for specific types of symptoms. One participant noted that budtenders “show you what’s best for you… it’s kind of like the doctor, but not really.” Participants expressed that budtenders listened to their feedback about their experiences with different products and used this to tailor suggestions. However, a few participants observed that advice might vary in quality, that “there are some clubs that are more knowledgeable,” and suggested to “go to a dispensary where they know what they’re talking about.”

#### Cannabis Retailer Staff as Caring and Nonjudgmental

Participants described feeling connected with their budtenders and generally viewed them as supportive of prenatal cannabis use. Many emphasized their loyalty to a particular retailer and expressed a sense of community. Several participants had worked in the cannabis industry themselves or had family members or friends in the industry. A few participants compared budtenders at cannabis retailers with the people they see on a regular basis at their local grocery store or bar; one participant noted “they all know me and my name” and another observed “they actually care about you.”

## Discussion

This qualitative study characterizes the perceptions of pregnant individuals on how legalization of cannabis for adult use has impacted prenatal cannabis use behaviors. Results complement data from epidemiologic studies indicating that the prevalence and frequency of prenatal cannabis use are increasing in the US^7,8^ and initial data indicating that legalization of cannabis for adult use is associated with increases in preconception and postpartum cannabis use.^20^ Participants perceived that legalization contributed to increases in prenatal cannabis use, due in part to widespread access to retailers and delivery services. There were mixed beliefs about cannabis marketing and advertising, with some seeing it as an annoyance that did not impact their desire to use, and others describing advertisements as a barrier to maintaining reduced frequency of use or abstinence.

Participants consistently described how legalization has contributed to greater acceptance of cannabis use in general and during pregnancy, resulting in increased prenatal use, reduced stigma, and greater willingness to discuss prenatal cannabis use with clinicians. While some participants continued to fear that a child could be removed from their home as a result of prenatal cannabis use, many described reduced concerns about involvement of CPS after legalization.

Participants trusted cannabis retailers to provide them with safer and improved products and access to knowledgeable, nonjudgmental, and caring budtenders. Most described the products available at retailers as safer than those sold on the illicit market (eg, not laced with other drugs and with fewer contaminants). Cannabis legally sold in retailers in California is reliably tested for issues like pesticide residues, heavy metals, and mold^28^ and is likely to be safer in this regard. However, testing does not ensure that products are safe, and recent studies indicate that cannabis products sold at retailers in California can have additives or contaminants with the potential for harm.^29,30^ Furthermore, the legal regulated market shares with illicit products the risks that arise from excessive tetrahydrocannabinol content or use of additives that are not necessarily safe for inhalation, such as certain added terpenes.

Participants also appreciated the ability to match different products and modes of administration with specific pregnancy-related concerns. Notably, one participant learned about the harms of prenatal blunt use from mandated tobacco warning labels after legalization; however, warning labels on use of cannabis products during pregnancy were not discussed. Finally, participants described feeling connected with budtenders from their preferred retailers, trusting them to provide expert, nonjudgmental advice about which products would help them most during pregnancy, which instilled a sense of community. We note that no differences were observed between Black and White perspectives related to the impacts of legalization.

### Implications

These findings suggest that pregnant individuals who use cannabis perceive that legalization of cannabis for adult use has contributed to increases in prenatal cannabis use and created new challenges and opportunities for supporting the health of pregnant individuals. Building on our key findings, we highlight potential interventions and research opportunities that can be further explored to address growing rates of prenatal cannabis use (Table 2).

**Table 2.** Potential Interventions and Research Opportunities to Leverage Legalization of Cannabis for Adult Use to Reduce Harms for Pregnant Individuals

Theme and subthemes from focus groups	Potential interventions and research opportunities^31^
**Greater access and exposure to cannabis**	
Retailer availability and delivery	Limit density of cannabis storefront retailers and delivery services Place caps on the number of retailers per inhabitant Mandate a required distance between retailers
Exposure to ubiquitous marketing and advertising	Increase advertising restrictions Test the effectiveness of requiring health warnings for prenatal cannabis use on cannabis advertisements, limiting advertisements (eg, banning all billboards), prohibiting discounts, and requiring plain packaging (eg, as done in Canada)^32^
**Increased acceptance**	
Reduced stigma, more discussions with health care practitioners, reduced concerns about CPS	Leverage reduced stigma and the willingness of a pregnant patient to discuss prenatal cannabis use with health care practitioners Screen pregnant patients for self-reported prenatal cannabis use Empower and invest in training obstetric clinicians to initiate conversations about prenatal cannabis use and provide education about health risks in a nonjudgmental, supportive manner Connect patients with alternative medications or supplements that have been proven safe for pregnancy-related symptoms (eg, morning sickness) Repeal punitive policies related to prenatal substance use^33^
**Trust in cannabis retailers**	
Trust in cannabis product safety	Alert consumers effectively about potential health risks of prenatal cannabis use Test the effectiveness of more prominent warnings on cannabis products Prohibit health and therapeutic claims on cannabis products related to cannabis use during pregnancy or while breastfeeding
Diverse product selection	Conduct research to understand the relative risks of different products and modes of use in pregnancy Seek a safer market of products Place stricter limits and taxes on high-potency cannabis products Prohibit cannabis products that are attractive to younger individuals (with the highest prevalence of prenatal cannabis use),^34^ including through flavors and imagery Clinicians should recommend and encourage abstinence while recognizing that patients have autonomy to make their own choices. If pregnant individuals choose to continue using cannabis, clinicians should encourage them to switch to safer products (eg, without tobacco) with potentially safer modes of administration (eg, from smoking to topicals).
Cannabis retailer staff as caring and nonjudgmental	Invest in training and education for budtenders and leverage their strong relationship with customers Conduct interviews and focus groups with budtenders to understand their willingness to talk with pregnant customers about cannabis Create educational materials and trainings for budtenders about the health risks associated with prenatal cannabis use and train them to share this information with customers Create educational materials and trainings to discourage budtenders from recommending cannabis use while pregnant or nursing and making therapeutic claims related to pregnancy symptoms (eg, nausea)

Our finding that greater access to legal cannabis is associated with prenatal cannabis use suggests that public health policies that have been successful in reducing risks associated with alcohol and tobacco,^35,36,37,38,39,40,41,42,43^ such as limiting density of retailers, may hold promise for reducing cannabis use during pregnancy. Furthermore, given that cannabis-related advertisements, billboards, and text messages were perceived as making it harder for some individuals to abstain from prenatal use, other strategies, such as required health warnings related to prenatal use on advertisements, prohibiting discounting, and limiting advertisements, should be tested to understand their potential to support pregnant individuals to maintain their abstinence or decreased use during pregnancy.

Importantly, participants’ perceptions of decreased stigma and greater willingness to disclose and discuss cannabis use with their clinicians after legalization should be leveraged. Obstetricians and other prenatal clinicians should be trained and empowered to screen for and initiate conversations about prenatal cannabis use, provide nonjudgmental information about the potential health risks, and link individuals who use cannabis with alternative medications or supplements that have been proven safe for pregnancy-related symptoms (eg, morning sickness). As noted in our focus groups, supportive and nonpenalizing patient-clinician conversations can be pivotal to helping pregnant individuals make more informed choices about whether to continue cannabis use during pregnancy.

The finding that many patients felt less concerned about potential CPS involvement following legalization is critically important, as it contributed to more honest discussions with health care practitioners about cannabis use. However, it is also noteworthy that some participants continued to express fears about CPS reporting. In California, even prior to legalization of cannabis for adult use, clinicians were not required to contact CPS or law enforcement when pregnant patients screened positive for cannabis use,^44^ and a positive toxicology test result at the time of delivery is not in and of itself a sufficient basis for reporting child abuse or neglect.^45^ However, despite recommendations from the American College of Obstetricians and Gynecologists to repeal punitive policies related to prenatal substance use,^33^ prenatal cannabis use is still included in definitions of child abuse or neglect that can lead to termination of parental rights in many states.^45^ This remains true even in some states that have legalized cannabis for adult use (eg, Colorado).^46^ This finding further highlights the need to reform antiquated policies in states that criminalize prenatal substance use,^33^ to avoid unintended negative public health consequences^47^ (eg, fewer patient-clinician discussions, missed opportunities for education and linkage to treatment).

Participants appreciated the diversification of cannabis products with different modes of administration and described improved quality and safety of products following legalization. Future studies are needed to test public health strategies that create a safer market (eg, placing stricter limits on high-potency products, prohibiting products attractive to young individuals who have the highest prevalence of prenatal cannabis use, and recommending purchasing from trusted sources) and to examine the health impacts of different cannabis products with different modes of administration.^31,32,33,34^

It is noteworthy that while one participant learned about the harms of blunt use during pregnancy as a result of mandated warnings on tobacco products and switched to use of a product that did not contain tobacco as a result, there was no discussion of the warning labels related to prenatal cannabis use. This suggests that the currently required small (size 6 font) warnings on cannabis products regarding prenatal cannabis use in California may be inadequate. As research on the health risks of different types and modes of cannabis use evolves, future studies that examine the effectiveness of posted in-store warnings or more prominent package warnings related to prenatal cannabis use are needed.

Pregnant participants described viewing budtenders as knowledgeable and trusted sources of nonjudgmental information, highlighting a unique opportunity to leverage the relationships between budtenders and customers to support future public health interventions. Future studies are needed to assess budtenders’ willingness to talk with pregnant customers about the potential harms of prenatal cannabis use. In California, budtender training is not required or offered by the Department of Cannabis Control,^48^ although a small percentage of local jurisdictions have training requirements.^49^ Training and educational materials are needed to increase budtenders’ awareness of the potential harms associated with prenatal cannabis use and to encourage a clear message that budtenders should avoid recommending prenatal cannabis use or making health or therapeutic claims related to cannabis use during pregnancy. At the very least, budtenders could be trained to steer pregnant individuals who choose to use cannabis toward products that do not contain tobacco and with potentially safer modes of administration (eg, topicals vs smoking). We note that participants did not differentiate medical vs adult-use retailers; however, in California, most retailers are licensed for both medical and adult use.^50^

### Limitations

This study has some limitations. Findings were limited to insured Black and White pregnant individuals seeking prenatal care in KPNC. Results may not be applicable to uninsured pregnant individuals, those with other races or ethnicities, or those living outside of California. We plan to explore similar themes in other racial and ethnic groups in future work and note that data from California can inform future recommendations in states that have not yet legalized cannabis. We prioritized recruitment of individuals with self-reported daily or weekly cannabis use in early pregnancy, and results may not be applicable to those who used less frequently or to those who chose not to disclose their use. Notably, while Black individuals were more likely to schedule a focus group than White individuals, they were less likely to show up or needed to reschedule and no additional focus group times worked. However, those who completed vs did not complete the focus groups did not differ on age, weeks pregnant, trimester at phone screening, frequency of prenatal cannabis use, or current use at recruitment. Additionally, perceptions of participants are based on focus groups at one time point after legalization and may not represent changes in behaviors or perceptions over time.

## Conclusions

This qualitative study addressed key gaps in understanding the impacts of cannabis legalization on pregnant individuals’ perceptions on cannabis use behaviors and beliefs. Results suggest that legalization may have contributed to increases in prenatal cannabis use and created both challenges and opportunities for supporting the health of pregnant individuals. Despite recommendations from national organizations that pregnant individuals should abstain from cannabis use during pregnancy,^2,5^ rates of prenatal cannabis use continue to rise in the US.^7^ The findings of this study could be leveraged to inform future recommendations and potential individual, clinician, and policy-based strategies to address increasing rates of prenatal cannabis use as legalization spreads across the US.

## Notes

Supplement.
Data Sharing Statement
