# Supplementary material for: Perceptions About Cannabis Following Legalization Among Pregnant Individuals With Prenatal Cannabis Use in California
Source: JAMA Netw Open. 2022 Dec 14;5(12):e2246912. doi: 10.1001/jamanetworkopen.2022.46912 (PMC9856570; doi:10.1001/jamanetworkopen.2022.46912)
Supplement: Supplement. — Data Sharing Statement [file jamanetwopen-e2246912-s001.pdf]

Data Sharing Statement

Young-Wolff. Perceptions About Cannabis Following Legalization Among Pregnant Individuals With Prenatal Cannabis Use in California. *JAMA Netw Open*. Published December 14, 2022. doi:10.1001/jamanetworkopen.2022.46912

Data
Data available: No
